# Sex difference in open-water swimming—The Triple Crown of Open Water Swimming 1875-2017

**DOI:** 10.1371/journal.pone.0202003

**Published:** 2018-08-29

**Authors:** Pantelis Theodoros Nikolaidis, Stefania Di Gangi, Caio Victor de Sousa, Fabio Valeri, Thomas Rosemann, Beat Knechtle

**Affiliations:** 1 Exercise Physiology Laboratory, Nikaia, Greece; 2 Institute of Primary Care, University of Zurich, Zurich, Switzerland; 3 Graduate Program in Physical Education, Catholic University of Brasilia, Brasilia, Brazil; 4 Medbase St. Gallen Am Vadianplatz, St. Gallen, Switzerland; Universita degli Studi di Verona, ITALY

## Abstract

The aim of the present study was to compare swimming performances of successful finishers of the 'Triple Crown of Open Water Swimming' from 1875 to 2017, assessing the effects of sex, the place of event and the nationality of swimmers. Data from 535 finishers in ‘Catalina Channel Swim’, 1,606 finishers in ‘English Channel Swim’ and 774 finishers in ‘Manhattan Island Marathon Swim’ were analysed. We performed different analyses and regression model fittings for all swimmers and annual top-5 finishers. Effects (sex, event, time, nationality) and interaction terms (event—time) were examined through a multi-variable spline mixed regression model. Considering all swimmers, we found that (i) women were approximately 0.06 km/h faster than men (p = 0.011) and (ii) Australians were 0.13 km/h faster than Americans (p = 0.004) and Americans were 0.19 km/h faster than British (p<0.001) and 0.21 km/h faster than Canadians (p = 0.015). When considering annual top-5 finishers, we found that (i) women were 0.07 km/h slower than men (p = 0.042) and (ii) Australians were not faster than Americans (p = 0.149) but Americans were 0.21 km/h faster than British (p<0.001). Our findings improved the knowledge about swim performances over time, in the three events, considering the effects of sex and the nationality of swimmers.

## Introduction

Open-water ultra-distance swimming is of increasing popularity. The number of athletes competing in channel [1, 2] and lake [3, 4] crossings increased in recent decades and the performance of the athletes improved [1, 4]. Especially, women reduced the gap to men considerably in long-distance swimming [3–5]. This decrease in sex difference in performance might be attributed to the increased participation of women in open-water swimming, which is associated with improved training and nutrition [4].

This trend was of great scientific interest as sex difference in performance is a major field in exercise physiology. Particularly, a debate has been arisen on whether women were going to outperform men in the future considering their larger rate of improvement compared to men [6, 7]. It has been assumed that women will outperform men in long-distance running in the near future [6]. Based upon small samples of female and male runners, it was assumed that women can outrun men in ultra-marathon running due to greater fatigue resistance [7]. However, the sex difference in ultra-distance running remained at ~12% when running distances up to 200 km were investigated [8].

The ‘Triple Crown of Open Water Swimming’ refers to open-water swimming's equivalent of the ‘Triple Crown of Thoroughbred Racing’ and includes two traditional Channel Crossings and a swim around Manhattan as the goal for open-water marathon swimmers (www.triplecrownofopenwaterswimming.com). These three events are the 33.7 km across the English Channel between England and France (‘English Channel Swim’), the 33 km across the Catalina Channel in Southern California, USA (‘Catalina Channel Swim’), and the 45.8 km around the Manhattan Island in New York, USA (‘Manhattan Island Marathon Swim’).

Recent studies found that women were faster than men in the ‘Catalina Channel Swim’ [9] and in the ‘Manhattan Island Marathon Swim’ [10], but not in the ‘English Channel Swim’ [1]. These findings might be due to the fact that both the ‘Catalina Channel Swim’ and the ‘Manhattan Island Marathon Swim’ are held in the USA but the ‘English Channel Swim’ in Europe between England and France and aspects of nationality of the participants might play an important role. For instance, it has been shown that US-Americans and Australians were among the fastest swimmers [11, 12]. Thus, superior performances would be expected in events that such nationalities had increased participation. Moreover, these studies investigated a limited sample of swimmers (*i*.*e*. top swimmers), but not the whole number of successful swimmers. Since the ‘English Channel Swim’ has more successful swimmers than both the ‘Manhattan Island Marathon Swim’ and the ‘Catalina Channel Swim’ and is held since 1875, the selection of top athletes might lead to false results.

The abovementioned studies have improved our understanding of sex differences in open-water swimming; however, they examined top swimmers instead of all swimmers (*i*.*e*. they analyzed the fastest finishers, *e*.*g*. top ten, instead of all the finishers in an event). Information about sex differences and the role of nationality considering all swimmers would be of great theoretical and practical value for exercise physiologists and coaches, respectively.

Therefore, the aim of the present study was to compare swimming performances of successful finishers in the three events of the ‘Triple Crown of Open Water Swimming’ in order to confirm or contradict the recent findings and to highlight potential selection biases. We expected that including all women and men in each event would lead to different findings for sex differences, compared to the analysis of only top swimmers, but conclusions about the effect of nationality on performance would still be valid. For this reason, we examined also the annual five fastest women and men in all the three events.

## Methods

### Ethics approval

All procedures used in the study were approved by the Institutional Review Board of Kanton St. Gallen, Switzerland, with a waiver of the requirement for informed consent of the participants given the fact that the study involved the analysis of publicly available data.

### Methodology

Swim times of all female and male solo swimmers, from 1875 to 2017, were obtained from the publicly available websites for the ‘Catalina Channel Swim’ (http://swimcatalina.com), the ‘Manhattan Island Marathon Swim’ (www.nycswim.org), and the ‘English Channel Swim’ (www.dover.uk.com/channel-swimming). Only solo swims were considered, i.e. relay swimmers and multiple crossings of a single swimmer were excluded. Swim times (h:min:s) were converted to swimming speed (km/h) to compare performance among the three different distances although different distances might affect swimming speed. In addition to swim times, event and year of competition, we collected: name—surname, sex and nationality of swimmers. We considered all finishers and the annual fastest top five separately. In one calendar year, one person could swim several times the same distance, but we deleted duplicate cases, when two observations had the same swim time.

### Statistical analysis

All statistical analyses were performed by the statistical package R, R Core Team (2016), Vienna, Austria, URL https://www.R-project.org/. Swimming speeds (km/h) were presented as mean (standard deviation) and categorical variables as number and percentage, N (%). Seventy non-missing nationalities were recorded, which were grouped into nine regions/countries: Africa, Asia, Australia (AUS), Canada, Central-South America, Europe except Great Britain (Rest of Europe), Great Britain (GBR), New Zealand (NZL) and USA. Great Britain was separated from the rest of Europe to better study the relationship between nationality and performance of British nationality in the ‘English Channel Swim’. Participation to each event, by nationality and during time, was compared between sexes using chi-square test for frequency distributions. We performed t-tests to compare the average speed between sexes in each event, then for the most prevalent countries and by period of time. In addition, effects (*i*.*e*. sex, event, time, nationality) and interactions (*i*.*e*. sex—time, event—time, event—sex, event-sex-time) were considered more rigorously through a spline regression model, with five degree of freedom basis splines in function of time. Time was defined as years from the median year. Since a swimmer might finish more than one race, we fitted a mixed model, with random effects on intercept for each unique swimmer. Different regression model specifications, with none, one, two and three term interactions were considered. Model selection was performed using both Akaike information criterion (AIC) and the Bayes information criterion (BIC).

The selected model was specified as follow:

Speed~[Fixedeffects(X)=Sex+Event:BS(cyear,df=5)+Nationality]+[Randomeffectsofintercept=Swimmers]

where *BS*(*cyear*, *df* = 5) denoted the 5 degree of freedom (*df*) basis splines and cyear denoted the year centered on the median. We performed two different analyses and we fitted two regression models: one for all swimmers and one for the annual top five finishers. For all tests and regressions, we defined statistical significance at p ≤ 0.05.

## Results

### Participation

Between 1875 and 2017, a total of 2,915 observations from 1,875 different finishers were considered, i.e. multiple finishes per swimmer were analyzed. The average finishes were 1.56 per swimmer, though only 454 (24%) swimmers have more than one record. The number of successful female and male solo finishers in ‘Catalina Channel Swim’, ‘English Channel Swim’ and ‘Manhattan Island Marathon Swim’ were 535, 1,606 and 774, respectively. The number of women was 553 (29% of the total unique swimmers) with 921 finishes (32%) and the number of men was 1,322 (71%) with 1,994 (68%) finishes.

The difference in sex distribution was presented in Table 1. Participation in each event and during time was different between females and males. Female participation had been always increasing and at a fastest rate compared with male participation. In the last period [2010, 2017], the participation was the highest representing 31.8% of the total female swimming speeds against 29.5% of the total male swimming speeds. The most prevalent countries in terms of finishers were USA, N = 1208 (41.4%), GBR, N = 657 (22.5%), rest of Europe, N = 285 (9.8%) and AUS, N = 317 (10.9%). The participation by nationality was different between females and males (p<0.001).

**Table 1:** All swimmers. Distribution of finishers by sex (F = female, M = male), event, region/country and average swimming speed comparison between sexes by event and the most prevalent regions/countries (USA, AUS, GBR, rest of Europe). Chi-square test p-values and t-test p-value (for average swimming speed) were reported. P-values were adjusted using Benjamini-Hochberg correction for multiple comparisons.

	Variable	Overall	F	M	p
**N**		2915	921	1994	
**Speed** **(mean (sd))**		3.47 (1.32)	3.53 (1.32)	3.44 (1.32)	0.080
**Event**					
**N (%)**	Catalina	535 (18.4)	194 (21.1)	341 (17.1)	0.031
	English	1606 (55.1)	497 (54.0)	1109 (55.6)	
	Manhattan	774 (26.6)	230 (25.0)	544 (27.3)	
**Period**					
**N (%)**					
	[1875,1960)	123 (4.2)	41 (4.5)	82 (4.1)	0.012
	[1960,1980)	302 (10.4)	74 (8.0)	228 (11.4)	
	[1980,1990)	505 (17.3)	140 (15.2)	365 (18.3)	
	[1990,2000)	520 (17.8)	171 (18.6)	349 (17.5)	
	[2000,2010)	583 (20.0)	202 (21.9)	381 (19.1)	
	[2010,2017]	882 (30.3)	293 (31.8)	589 (29.5)	
**Nationality** Group* **N (%)**					
	Africa	82 (2.8)	10 (1.1)	72 (3.6)	<0.001
	Asia	131 (4.5)	12 (1.3)	119 (6.0)	
	AUS	317 (10.9)	115 (12.5)	202 (10.1)	
	Canada	72 (2.5)	35 (3.8)	37 (1.9)	
	Central-South America	130 (4.5)	33 (3.6)	97 (4.9)	
	GBR	657 (22.5)	223 (24.2)	434 (21.8)	
	Rest of Europe	285 (9.8)	87 (9.4)	198 (9.9)	
	NZL	33 (1.1)	14 (1.5)	19 (1.0)	
	USA	1208 (41.4)	392 (42.6)	816 (40.9)	
**Overall**			**Speed Mean (sd)**		
	Event		F	M	p
	Catalina		2.86 (0.58)	2.84 (0.58)	0.679
	English		2.85 (0.59)	2.65 (0.56)	<0.001
	Manhattan		5.56 (0.68)	5.41 (0.46)	<0.001
**Nationality**					
USA			Speed Mean (sd) N		
	Event		F	M	p
	Catalina		2.87 (0.60) N = 137	2.89 (0.57) N = 227	0.788
	English		2.89 (0.54) N = 95	2.71 (0.54) N = 187	0.021
	Manhattan		5.54 (0.69) N = 160	5.41 (0.46) N = 402	0.024
AUS			Speed Mean (sd) N		
	Event		F	M	p
	Catalina		3.13 (0.56) N = 13	3.01 (0.42) N = 21	0.505
	English		3.12 (0.54) N = 76	2.77 (0.51) N = 152	<0.001
	Manhattan		6.00 (0.68) N = 26	5.58 (0.41) N = 29	0.010
GBR			Speed Mean (sd) N		
	Event		F	M	p
	Catalina		2.56 (0.57) N = 13	2.68 (0.37) N = 19	0.472
	English		2.69 (0.57) N = 185	2.47 (0.47) N = 376	<0.001
	Manhattan		5.38 (0.40) N = 25	5.26 (0.42) N = 39	0.388
Rest of Europe			Speed Mean (sd) N		
	Event		F	M	p
	Catalina		2.97 (0.41) N = 16	2.79 (0.66) N = 24	0.374
	English		2.90 (0.67) N = 65	2.77 (0.70) N = 149	0.374
	Manhattan		5.17 (1.17) N = 6	5.42 (0.43) N = 25	0.374

* Using International Olympic Committee (IOC) country codes (https://www.olympic.org/), we defined nationality group as Africa [5 countries, international codes: EGY, NAM, RSA, TUN, ZIM], Asia [15 countries: BAN, CHN, IND, IRN, IRQ, ISR, JOR, JPN, KOR, KSA, LIB, MAS, PAK, SYR, TUR], Australia (AUS), Canada, Central-South America [11 countries: ARG, BAR, BRA, CHI, CUB, DOM, ECU, GUA, MEX, URU, VEN], Europe except Great Britain [33 countries: AUT, BEL, BUL, CRO, CYP, CZE, DEN, ESP, EST, FIN, FRA, GER, GRE, HUN, IRL, ISL, ITA, JUG, LUX, MDA, MKD, MLT, NED, NOR, POL, POR, ROU, RUS, SLO, SRB, SUI, SVK, SWE], Great Britain (GBR, or GRB—including Scotland and Wales), New Zealand (NZL including VAN) and United States of America (USA).

### Performance considering all swimmers

The (kernel estimate) density curves of the observed swimming speeds by sex and event were presented in S1 Fig. We tested means (t-test) between sexes for each event and for the most prevalent regions/countries (Table 1). Except ‘Catalina Channel Swim’ (females 2.86 km/h (0.58) *versus* males 2.84 km/h (0.58), p = 0.679), there were differences between females and males average swimming speeds in each event: ‘English Channel Swim’, females 2.85 km/h (0.59) *versus* males 2.65 km/h (0.56), p<0.001, and ‘Manhattan Island Marathon Swim’, females 5.56 km/h (0.68) *versus* males 5.41 km/h (0.46) p<0.001. For both sexes, swimming speed average in ‘Manhattan Island Marathon Swim’ was faster than in ‘Catalina Channel Swim’ and swimming speed average in both events was faster than in ‘English Channel Swim’.

**Fig 1:**
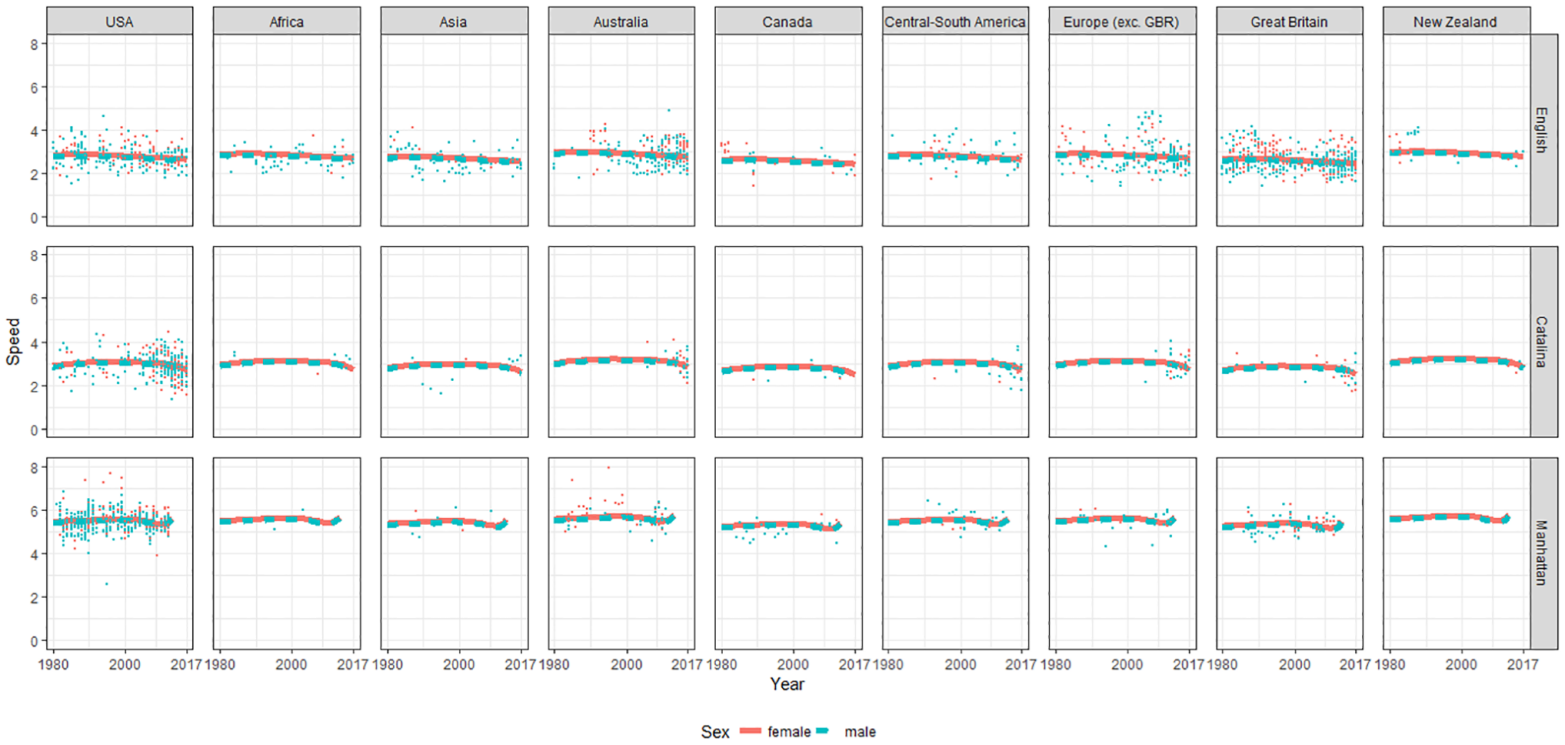
Observed and predicted swimming speeds during time by sex, event and nationality for all swimmers.

Predicted values (lines) and observed swimming speeds (points) during time, from 1980 onward, by event, nationality and sex were plotted in Fig 1. Predicted values were computed according to the spline regression model whose details, including model selection criteria were presented in Table 2. In the model, all observations from 1875 were considered, however, only results from 1980 onward were included in Fig 1 and Table 3 in order to better highlight the trends in the last four decades. In Table 2, model 5, with three-term (sex-time-event) interaction, compared to the model selected, with (event-time) interaction, had a slightly lower AIC but a higher BIC. Therefore, the selected reduced model, nearly matched, or in some cases outfitted the full model, which would be quite tricky to interpret. At the same level of calendar year, nationality and event, men were near 0.06 km/h slower than women (estimate = -0.06478, p = 0.011) (Table 2, Fig 1).

**Table 2:** Estimates, standard errors and p-value of spline regression model, 5 degree of, freedom, BS = basis spline. Year was defined as difference from 2000 (median year).

	All Swimmers N = 2915, swimmers = 1875			Annual Top Five N = 1150, swimmers = 741		
	Estimate	Std. Error	p	Estimate	Std. Error	p
(Intercept)	2.17528	0.49178	<0.001	2.23029	0.47841	<0.001
**Event (ref English)**						
Catalina	4.82923	3.45928	0.163	12.97047	4.04597	0.001
Manhattan	-3.55725	3.43766	0.301	-7.84694	3.61623	0.030
**Sex (ref F)**						
sex: M	-0.06478	0.02541	0.011	0.07157	0.03509	0.042
**Year**						
BS (year) 1	-1.38662	0.71596	0.053	-1.68518	0.72951	0.021
BS (year) 2	1.13244	0.46724	0.015	0.67635	0.46253	0.144
BS (year) 3	0.58831	0.50243	0.242	1.11964	0.49446	0.024
BS (year) 4	0.52664	0.48915	0.282	1.07132	0.48038	0.026
BS (year) 5	0.48071	0.49436	0.331	1.06046	0.48906	0.030
**Nationality (ref USA)**				Other:		
				-0.0002	0.04675	0.997
Africa	0.04819	0.06459	0.456			
Asia	-0.08772	0.05684	0.123			
Australia (AUS)	0.12709	0.04371	0.004	0.09928	0.06873	0.149
Canada	-0.20771	0.08563	0.015			
Central-South America	-0.00009	0.05913	0.999			
Europe (except GBR)	0.04991	0.04015	0.214			
Great Britain (GBR)	-0.18821	0.03374	<0.001	-0.20791	0.05514	<0.001
New Zealand (NZL)	0.14882	0.11714	0.204			
**Event: Year**						
Event Catalina: BS (year) 1	-6.67685	4.63568	0.150	-18.13887	5.40415	0.001
Event Manhattan: BS (year) 1	8.68792	4.93764	0.079	15.00808	5.09649	0.003
Event Catalina: BS (year) 2	-4.81426	3.18463	0.131	-11.55978	3.7214	0.002
Event Manhattan: BS (year) 2	5.34044	3.22401	0.098	9.13077	3.43271	0.008
Event Catalina: BS (year) 3	-4.5313	3.50688	0.196	-13.73699	4.12406	0.001
Event Manhattan: BS (year) 3	6.50976	3.47768	0.061	10.74124	3.66311	0.003
Event Catalina: BS (year) 4	-4.54717	3.44131	0.186	-12.56461	4.00609	0.002
Event Manhattan: BS (year) 4	5.9362	3.40622	0.082	9.46632	3.58107	0.008
Event Catalina: BS (year) 5	-4.79796	3.46496	0.166	-13.02151	4.05953	0.001
Event Manhattan: BS (year) 5	8.69372	3.82631	0.023	11.9961	3.75297	0.001
		AIC	BIC		AIC	BIC
		3969.3	4142.6		1490.6	1611.7
Alternative (reduced) models		AIC	BIC		AIC	BIC
model 1 no interactions		3990.4	4103.9		1531.4	1602.0
model 2 interaction Sex:Event		3993.8	4119.3		1529.9	1610.7
model 3 interaction Sex:BS(year)		3992.4	4135.9		1536.0	1631.9
model 4 interaction Sex:BS (year)+Event:BS(year)		3971.8	4175.0		1492.6	1639.0
model 5 interaction Sex:BS (year): Event		3965.3	4240.3		1448.4	1655.4

**Table 3:** Estimated values of swimming speed by event, sex and year.

**All finishers—estimated difference M—F range (-0.06, -0.06)**				
**English**	**Period of time**			
	[1980,1990)	[1990,2000)	[2000,2010)	[2010,2017]
Estimated F (min, max)	(2.65, 3.03)	(2.62, 3.03)	(2.53, 2.97)	(2.45, 2.88)
Estimated M (min, max)	(2.58, 2.97)	(2.56, 2.96)	(2.47, 2.90)	(2.38, 2.82)
**Catalina**	**Period of time**			
	[1980,1990)	[1990,2000)	[2000,2010)	[2010,2017]
Estimated F (min, max)	(2.70, 3.20)	(2.85, 3.23)	(2.82, 3.23)	(2.48, 3.16)
Estimated M (min, max)	(2.63, 3.13)	(2.79, 3.17)	(2.75, 3.16)	(2.41, 3.10)
**Manhattan**	**Period of time**			
	[1980,1990)	[1990,2000)	[2000,2010)	[2010,2017]
Estimated F (min, max)	(5.27, 5.68)	(5.34, 5.75)	(5.17, 5.74)	(5.15, 5.79)
Estimated M (min, max)	(5.20, 5.62)	(5.27, 5.68)	(5.11, 5.68)	(5.08, 5.73)
**Annual top five swimmers—estimated difference M—F range (0.07, 0.07)**				
**English**	**Period of time**			
	[1980,1990)	[1990,2000)	[2000,2010)	[2010,2017]
Estimated F (min, max)	(2.87, 3.32)	(3.02, 3.40)	(3.10, 3.42)	(3.08, 3.41)
Estimated M (min, max)	(2.94, 3.39)	(3.09, 3.48)	(3.17, 3.49)	(3.15, 3.48)
**Catalina**	**Period of time**			
	[1980,1990)	[1990,2000)	[2000,2010)	[2010,2017]
Estimated F (min, max)	(2.75, 3.09)	(2.68, 3.04)	(2.72, 3.44)	(3.03, 3.53)
Estimated M (min, max)	(2.82, 3.17)	(2.75, 3.11)	(2.79, 3.51)	(3.10, 3.60)
**Manhattan**	**Period of time**			
	[1980,1990)	[1990,2000)	[2000,2010)	[2010,2017]
Estimated F (min, max)	(5.31, 5.84)	(5.57, 5.98)	(5.33, 5.94)	(5.40, 6.13)
Estimated M (min, max)	(5.39, 5.92)	(5.64, 6.05)	(5.40, 6.01)	(5.47, 6.20)

Therefore, we observed an effect of sex in the intercept of the model. Event was not statistically significant alone but in interaction with time (the last term of interaction ‘Manhattan Island Marathon Swim’: BS (Year) 5 had p = 0.023). Accordingly, an effect of event in the slope of the model was shown. As presented in Fig 1, the trend of performance over time was decreasing in ‘English Channel Swim’ and in ‘Catalina Channel Swim’, but in the latter the trend was not monotonic. On the contrary, in ‘Manhattan Island Marathon Swim’, the trend was increasing but not monotonically. Regarding nationality, Australian swimmers were near 0.13 km/h faster than Americans (estimate = 0.12709, p = 0.004). Instead, British (estimate = -0.18821, p<0.001) and swimmers from Canadians (estimate = -0.20771, p = 0.015) were slower than Americans.

The range (min, max) of yearly estimated values of swimming speeds by sex, for each period of time from 1980 onward and for each event, was reported in Table 3. That is, the minimum and maximum values of 10 fitted years, except the last period of 7 years, were presented for each period, event and sex. Because the interaction terms (sex-event) and (sex-time) were not considered in the model, the range of the estimated difference between sexes, men range—women range, was constant (-0.06, -0.06) across event and time. In the ‘English Channel Swim’, the differences between maximum and minimum of the estimated values of each sex were the smallest, and the minimum and maximum reduced over time. In the ‘Catalina Channel Swim’, the minimum and maximum increased until 2000, and then decreased. On the contrary, the differences between maximum and minimum of each sex decreased until 2000 and then increased. In the ‘Manhattan Island Marathon Swim’, the minimum and maximum increased during the first period then decreased and the maximum increased again in the last period. The differences of minimum and maximum instead, after first decreasing, increased. In particular, the range of estimated male performance in period [1990, 2000] was equal to the range of estimated female performance one decade earlier, in period [1980, 1990].

To compare estimated values (Table 3) with observed values, the average swimming speed comparison between sexes by event and period of time was reported (S1 Table). P-values of mean t-tests were presented in order to refine the descriptive part of the analysis and to provide an overview of the interaction effects that were not considered in our statistical model. Moreover, details of average performance before 1980 were also provided. In particular, in ‘Catalina Channel Swim’, no differences between females and males during time were found. In ‘Manhattan Island Marathon Swim’, females were slower only in period [1875,1960) compared to males. Average performance, from 1980 onward, for both sexes, after increasing over time had overall decreased from 1980 to 2017 for all events except for men in ‘Manhattan Island Marathon Swim’.

### Performance considering the annual top five swimmers

The total number of observations for annual top five swimmers was 1150 (741 swimmers). The number of women was 299 (40%) with 506 observations (44% of the total observations) and the number of men was 442 (60%) with 644 (56% of the total observations). The (kernel estimate) density curves of the observed swimming speeds, by sex and event was plotted in S1 Fig. Considering the annual top five swimmers, the distribution of women, in particular with regards to its skewness, was more similar to the distribution of men, compared with all swimmers.

Participation and average swimming speeds for each event and the most prevalent nationalities (USA, AUS, GBR, and rest of Europe) were presented in Table 4. The participation did not change with event (p = 0.286) and with time (p = 0.071), but changed with nationality (p<0.001). No differences between females and males for average swimming speeds in each event were found and in each event for the most prevalent nationalities.

**Table 4:** Annual top five swimmers. Distribution of finishers by sex (F = female, M = male), event, region/country and average swimming speed comparison between sexes by event and the most prevalent regions/countries (USA, AUS, GBR, rest of Europe). Chi-square test p-values and t-test p-value (for average swimming speed) were reported. P-values were adjusted using Benjamini-Hochberg correction for multiple comparisons.

	Variable	F	M	p
**N**	1150	506	644	
**Speed** **(mean (sd))**		3.84 (1.34)	3.74 (1.32)	0.201
**Event**				
**N (%)**	Catalina	106 (20.9)	158 (24.5)	0.286
	English	259 (51.2)	325 (50.5)	
	Manhattan	141 (27.9)	161 (25.0)	
**Period N (%)**				
	[1875,1960)	41 (8.1)	67 (10.4)	0.071
	[1960,1980)	61 (12.1)	111 (17.2)	
	[1980,1990)	94 (18.6)	112 (17.4)	
	[1990,2000)	96 (19.0)	124 (19.3)	
	[2000,2010)	121 (23.9)	133 (20.7)	
	[2010,2017]	93 (18.4)	97 (15.1)	
**Nationality group N (%)**				
	Africa	6 (1.2)	32 (5.0)	<0.001
	Asia	6 (1.2)	45 (7.0)	
	AUS	69 (13.6)	54 (8.4)	
	Canada	21 (4.2)	9 (1.4)	
	Central-South America	10 (2.0)	40 (6.2)	
	GBR	102 (20.2)	106 (16.5)	
	Rest of Europe	58 (11.5)	60 (9.3)	
	NZL	6 (1.2)	11 (1.7)	
	USA	228 (45.1)	287 (44.6)	
**Overall**		**Speed Mean (sd)**		
	Event	F	M	p
	Catalina	3.11 (0.58)	3.02 (0.70)	0.846
	English	3.11 (0.58)	3.10 (0.68)	0.924
	Manhattan	5.74 (0.72)	5.75 (0.43)	0.924
**Nationality**				
USA		Speed Mean (sd) N		
	Event	F	M	p
	Catalina	3.11 (0.63) N = 77	3.09 (0.65) N = 118	0.850
	English	3.11 (0.56) N = 49	3.18 (0.65) N = 47	0.850
	Manhattan	5.72 (0.71) N = 102	5.74 (0.46) N = 122	0.850
AUS		Speed Mean (sd) N		
	Event	F	M	p
	Catalina	3.39 (0.40) N = 9	3.37 (0.31) N = 7	0.948
	English	3.46 (0.40) N = 39	3.36 (0.45) N = 37	0.523
	Manhattan	6.14 (0.63) N = 21	5.94 (0.25) N = 10	0.523
GBR		Speed Mean (sd) N		
	Event	F	M	p
	Catalina	3.27 (0.23) N = 3	2.89 (0.54) N = 4	0.310
	English	2.94 (0.59) N = 90	2.73 (0.65) N = 95	0.069
	Manhattan	5.40 (0.27) N = 9	5.71 (0.45) N = 7	0.162
Rest of Europe		Speed Mean (sd) N		
	Event	F	M	p
	Catalina	3.08 (0.39) N = 12	3.00 (1.11) N = 7	0.821
	English	3.12 (0.68) N = 42	3.44 (0.79) N = 47	0.132
	Manhattan	5.09 (1.50) N = 4	5.77 (0.17) N = 6	0.433

**Fig 2:**
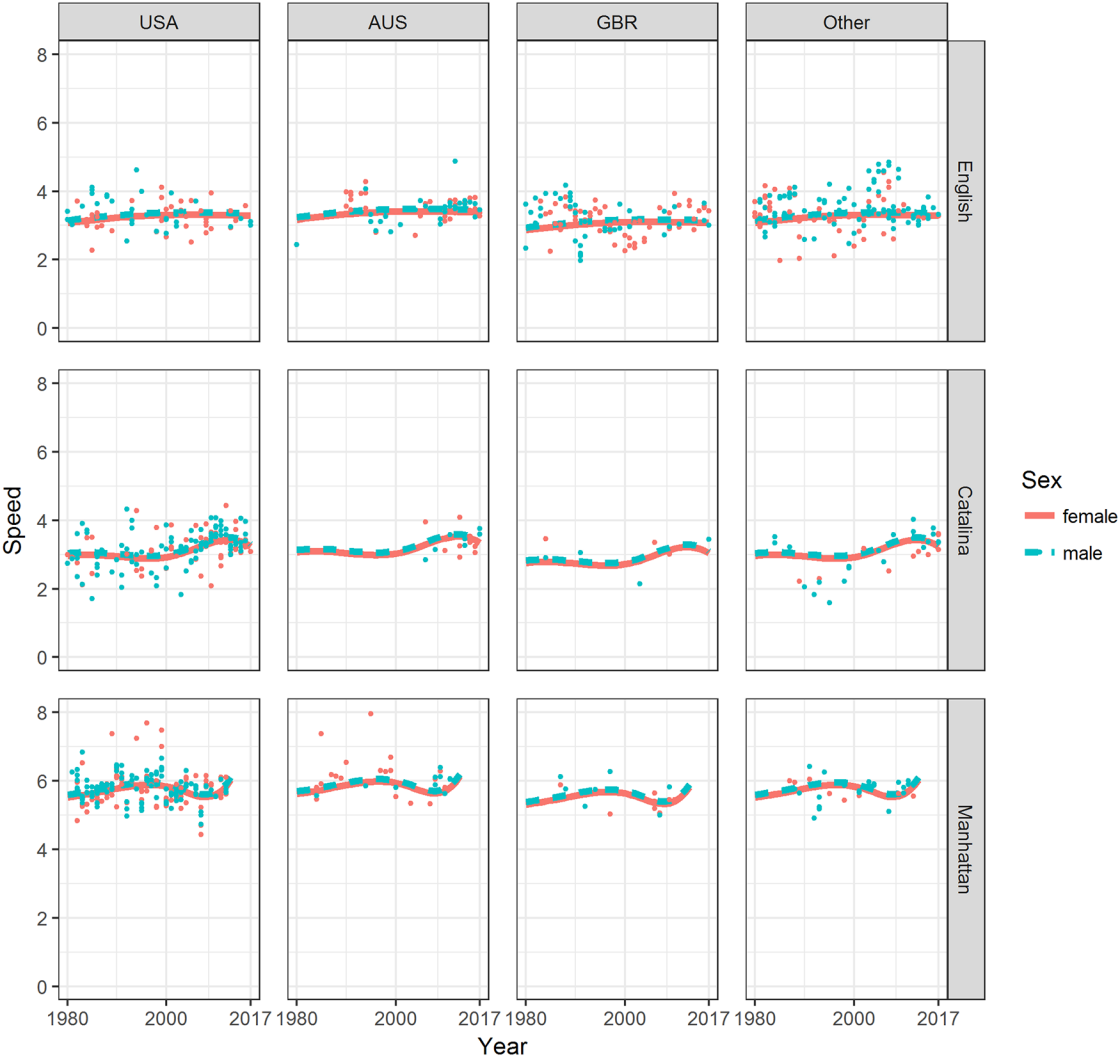
Observed and predicted swimming speeds during time by sex, event and nationality for annual top five swimmers.

Predicted values (lines) and observed swimming speeds (points) for each sex during time, from 1980 onward, by sex, event and nationality were plotted in Fig 2. Predicted values were computed according to the spline regression model whose details, including model selection criteria were provided in Table 2. In the model, all observations from 1875 were considered, but only results from 1980 onward were shown in Fig 2 and Table 3 in order to better highlight trends in the last four decades. In Table 2, it was shown that the selected model—with interaction (event-time)—was the best trade-off between the full complex model, with the lowest AIC, and a more parsimonious model with a lower BIC. Men were by near 0.07 km/h faster than women (estimate = 0.07157, p = 0.042) at the same level of calendar year, nationality and event (Table 2, Fig 2). Therefore, an effect of sex in the intercept of the model was shown. Event was statistically significant alone and in interaction with time. Therefore, effects of event in both slope and intercept of the model were found. The trend over time was overall increasing, but not monotonically, for all of the three events (Fig 2). Regarding nationality, Australians were not faster than Americans (p = 0.149), but swimmer from Great Britain were near 0.21 km/h slower than Americans (estimate = -0.20791, p<0.001).

The range (min, max) of yearly estimated values of swimming speeds by sex for each time period from 1980 onward was shown in Table 3. That is, for each period, event and sex, the minimum and maximum values of 10 fitted years, except the last period of 7 years, were reported. Because the interaction terms (sex-event) and (sex-time) was not considered in our model, the range of the estimated difference between sexes, men range—women range, was constant (0.07, 0.07) across event and time. The differences between maximum and minimum of the estimated values of both sexes were the smallest, and the minimum and maximum increased until 2010 and then decreased in the ‘English Channel Swim’. In the ‘Catalina Channel Swim’, instead, the minimum and maximum decreased until 2000, and then increased. Furthermore, the differences between minimum and maximum increased until 2010 and then increased. In the ‘Manhattan Island Marathon Swim’, the minimum and maximum increased during the first period then decreased and increased again in the last period. The differences between minimum and maximum, after first decreasing, increased.

## Discussion

The main findings of the present study were that (*i*) the participation in women and men varied by nationality and for all swimmers also by event, (*ii*) the nationality of finishers varied by event, (*iii*) women were faster than men when considering all swimmers; on the contrary, men were faster than women when considering annual top five, (*iv*) swimming speed was the fastest in the ‘Manhattan Island Marathon Swim’ and was the slowest for all swimmers in the ‘English Channel Swim’, and (*v*) Australians were faster than Americans, who in turn were faster than British and Canadians (all swimmers, mixed model).

### The participation in women and men varied by nationality

A first important finding was that female participation varied by nationality. Australian swimmers have a different approach to open-water ultra-distance swimming events than female swimmers from the United States of America and Great Britain. In pool-swimming at world class level, swimmers from Australia were more consistent than those from the United States and other nations when the relationship between world-ranking and performance at the Olympic Games was investigated [12].

In open-water swimming events, the rates of participation of women and men vary by race distance and event. In these solo swims with a partially very long history, the participation in women and men varied by nationality. However, no dominance of a particular nationality for all race distances was observed in the FINA (Fédération Internationale de Natation) races in 5 km, 10 km and 25 km held between 2000 and 2016 [13]. In these races, women and men compete together in partially very large fields where, interestingly, men were always faster than women although the possibility of drafting exists. In solo swims like the open-water swimming events of the ‘Triple Crown of Open Water Swimming’, drafting is not possible. Potential explanations could be that swimmers competing in FINA World Cup Races are elite swimmers also competing at World Championships and Olympic Games whereas swimmers competing in the events of the ‘Triple Crown of Open Water Swimming’ are mostly recreational athletes. Therefore, the motivation for swimmers competing in the FINA races seems different since these races offer prize money [https://swimswam.com/prize-money-2017-fina-world-championships-remains-unchanged/] whereas no prize money can be earned in the events of the ‘Triple Crown of Open Water Swimming’.

### The nationality of finishers varied by event

Considering the variation of the nationality of finishers by event, more US-American swimmers competed in the ‘Catalina Channel Swim’ and in the ‘Manhattan Island Marathon Swim’ and more British swimmers in the ‘English Channel Swim’. This is most likely due to the fact that travelling for US-Americans to Europe seems more costly than travelling within the own country. Similarly, it would be more affordable for British swimmers to travel to the ‘English Channel’ than to fly over the Atlantic to the United States of America.

In the FINA races held worldwide between 2000 and 2016 in 5 km, 10 km and 25 km, swimmers preferred races held on the continent where they lived. Europeans were the most finishers in races held in Europe, and Americans finished most in races held in America. Also, relatively more Asians finished in races held in Asia than on the other continents. Nationality played a role, not only for performance and participation, but also on the prevalence of non-finishers of the 10 km and the 25 km races [13].

### Sex differences in swimming speed

Regarding the summary statistics, women were faster than men, in the ‘English Channel Swim’ and in the ‘Manhattan Island Marathon Swim’ when all swimmers were considered. This could be also influenced by the difference in sex participation. In fact the overall men-to-women ratio was the lowest (1.76) in ‘Catalina Channel Swim’ compared with 2.23 in the ‘English Channel Swim’ and 2.37 in the ‘Manhattan Island Marathon Swim’.

This could be explained by the fact that more casual women swimmers enrolled in ‘Catalina Channel Swim’, which slowered the overall average time for women. When the annual top five were considered, the men-to-women ratio was close to 1 and did not change by event. Moreover, in the ‘Catalina Channel Swim’ nationality did not vary by sex.

However, after correcting for nationality, repeated measurements within swimmers and interaction terms event-time for all swimmers, women were faster than men, but, on the contrary, men were faster than women when considering annual top five swimmers. These results support recent findings for the ‘Catalina Channel Swim’ [9] and the ‘Manhattan Island Marathon Swim’ [10]. However, when all women and men were considered in the ‘English Channel Swim’, findings of existing studies could not be confirmed where men were faster than women [1, 14].

When the annual five fastest swimmers were considered, we found no differences in swimming speed between women and men examining the summary statistics for the three events and all prevalent nationalities. This finding is in contrast to the FINA races where men were faster than women with a similar sex difference for all three distances [13] but in the analysis of the FINA races, all women were compared to all men. However, after correcting for nationality, repeated measurements within swimmers and interaction terms: event-time, our findings for annual top five are in line with the FINA races [13].

### Swimming speed was the fastest in ‘Manhattan Island Marathon Swim’

A further finding was that swimming speed was the fastest in ‘Manhattan Island Marathon Swim’ although the event was the longest with 45.8 km around the Manhattan Island in New York compared to 33.7 km across the English Channel between England and France and the 33 km across the Catalina Channel in Southern California. The faster swimming speed in ‘Manhattan Island Marathon Swim’ compared to the other two events is explained by the current of the Hudson River and the tides (http://blog.marathonswimmers.org/2011/06/tides-are-everything). In the ‘English Channel Swim’ and in the ‘Catalina Channel Swim’, swimmers have to swim against currents. In the English Channel, also tides can prevent swimmers from achieving fast swim times (www.bbc.com/news/uk-england-kent-10782301).

### Australians were faster than Americans, British and others

An important finding was that both female and male swimmers from Australia were the fastest. Based upon finding for triathletes competing in ‘Ironman Hawaii’ one might assume that mainly athletes from the local region where the events are held would participate to them and would also be the fastest [15]. Indeed, in the ‘English Channel Swim’ between 1875 and 2013, mainly British swimmers participated and were also among the fastest [2].

However, in the present analysis, female swimmers from Australia were the fastest in all three events although these events were held in the United States of America and in Europe. It is well known that swimmers originating from Australia are among the fastest together with US-American swimmers in pool swimming at world class level events such as the Olympic Games [11, 12].

Australia was placed third in the Rio 2016 Olympic Games aquatics medals and second in the swimming events (www.fina.org/event/xxxi-olympic-games/medalsm). Australia was placed eighth in the London 2012 Olympic Games aquatics medals, third according to the total number of medals (www.swimming.org.au). Based upon the actual findings, Australians might also be the best in open-water ultra-distance swimming.

### Limitations, strength, implications for future research and practical applications

Considering the differences among the three events, it might be highlighted that the findings of the present study were event-specific and should be generalized to other ultra-distance swimming races with caution. The greater limitation of this study was that information about the age of each swimmer was not available. For this reason, our current model might not be properly specified. Moreover, interaction between sex and nationality was not considered in the regression model because, for some region/country, the number of observations was small. On the other hand, a reduced model with only one interaction term (event-time) was used. For this reason, we found that sex differences did not change with time and event. AIC criterion suggested, as an alternative model, a three-way interaction sex-event-time but interpreting this model and identifying the global effect of each predictor would not have been immediate. Strength of this study was that, in contrast to previous research that examined a limited sample of swimmers (*i*.*e*. top swimmers) [1, 9, 10], it adopted a novel approach by investigating all finishers. Future studies might investigate the sex difference in performance in the FINA (Fédération Internationale de Natation) for the official World Cup races held over 5 km, 10 km and 25 km [16–18]. Based upon the actual findings, women should also be faster than men in these races. The results provided practical information for coaches and swimmers on important aspects of performance so they could optimize their preparation for such races. This was of great practical value especially considering that open-water swimming rapidly grew in popularity [19].

## Conclusions

In our statistical modeling framework, women were faster than men, and Australians were faster than Americans, who were faster than British and Canadians. When the annual top five swimmers were considered, men were faster than women, and Americans were faster than British.

## Supporting information

S1 Fig Estimated kernel density of observed swimming speeds by sex and event, for all swimmers and annual top five swimmers. (TIFF)

S1 Table All swimmers. **Females and males comparison in average swimming speeds by period of time and event**. P-values were adjusted using Benjamini-Hochberg correction for multiple comparisons.
(DOCX)

S1 Dataset All swimmers dataset. (XLS)
