# Supplementary figures and images for: Sex difference in open-water swimming—The Triple Crown of Open Water Swimming 1875-2017
Source: PLoS One. 2018 Aug 29;13(8):e0202003. doi: 10.1371/journal.pone.0202003 (PMC6114520; doi:10.1371/journal.pone.0202003)

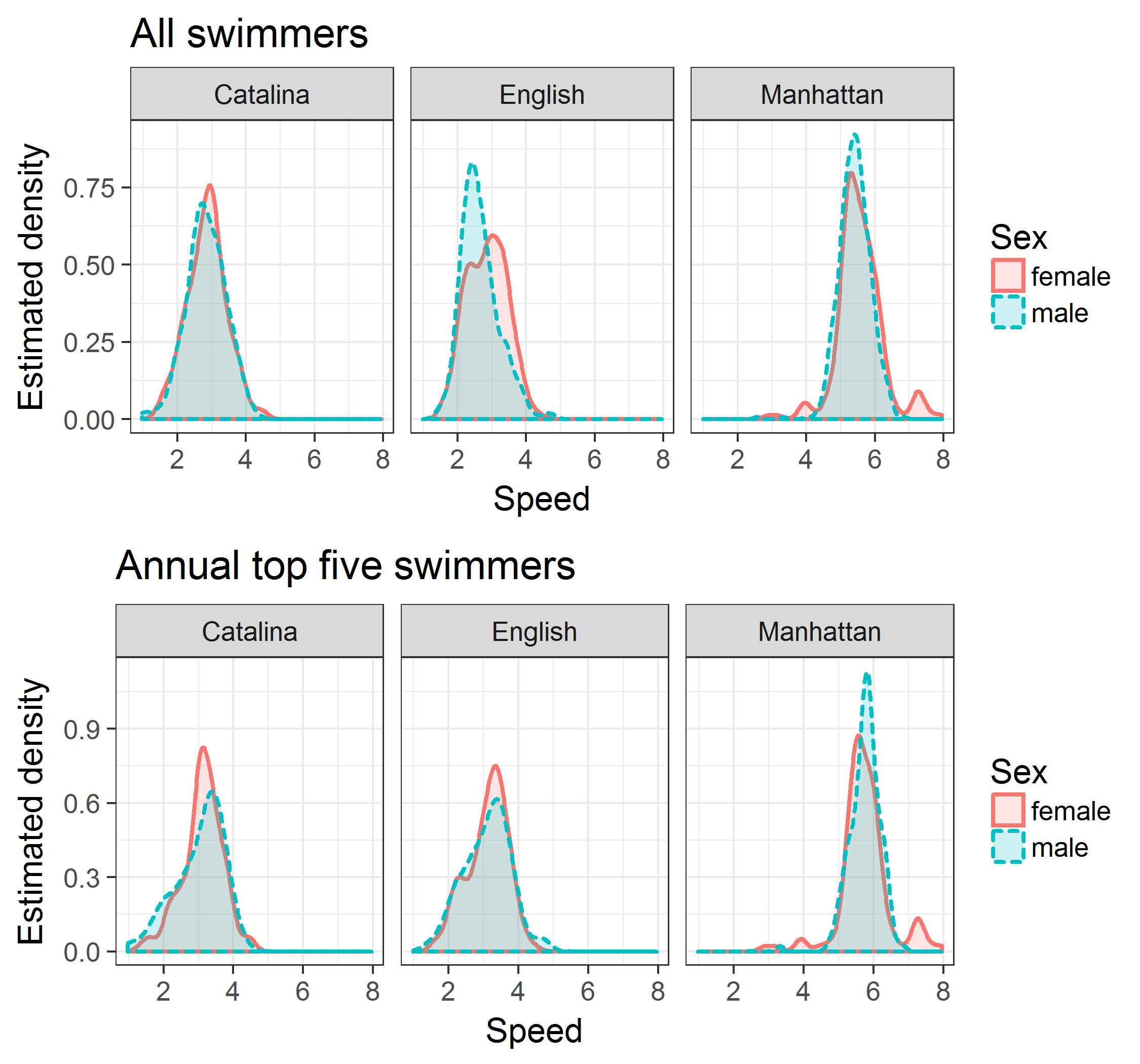

Supplement: S1 Fig — (TIFF) [file pone.0202003.s001.tiff]
