# Supplementary material for: Sex difference in open-water swimming—The Triple Crown of Open Water Swimming 1875-2017
Source: PLoS One. 2018 Aug 29;13(8):e0202003. doi: 10.1371/journal.pone.0202003 (PMC6114520; doi:10.1371/journal.pone.0202003)
Supplement: S1 Table — Females and males comparison in average swimming speeds by period of time and event. P-values were adjusted using Benjamini-Hochberg correction for multiple comparisons. (DOCX) [file pone.0202003.s002.docx]

**S1 Table. All swimmers. Females and males comparison in average swimming speeds by period of time and event**. P-values were adjusted using Benjamini-Hochberg correction for multiple comparisons.

| Overall |  | Speed Mean (sd) N | |  |
| --- | --- | --- | --- | --- |
|  | Period of time | F | M | p |
|  | [1875,1960) | 2.46 (0.71) N=41 | 2.43 (0.97) N=82 | 0.996 |
|  | [1960,1980) | 2.91 (0.75) N=74 | 2.62 (0.55) N=228 | <0.001 |
|  | [1980,1990) | 3.96 (1.35) N=140 | 3.96 (1.35) N=365 | 0.996 |
|  | [1990,2000) | 4.02 (1.58) N=171 | 4.00 (1.57) N=349 | 0.996 |
|  | [2000,2010) | 3.89 (1.35) N=202 | 3.75 (1.34) N=381 | 0.708 |
|  | [2010,2017] | 3.09 (0.96) N=293 | 3.03 (0.97) N=589 | 0.722 |
| Catalina |  | Speed Mean (sd) N | |  |
|  | Period of time | F | M | p |
|  | [1875,1960)^1^ | 2.14 (0.55) N=10 | 1.89 (0.60) N=19 | 0.471 |
|  | [1960,1980) | 3.11 (0.65) N=14 | 3.24 (0.58) N=16 | 0.670 |
|  | [1980,1990) | 2.89 (0.51) N=10 | 2.91 (0.56) N=22 | 0.936 |
|  | [1990,2000) | 3.01 (0.67) N=9 | 2.74 (0.68) N=24 | 0.471 |
|  | [2000,2010) | 3.12 (0.46) N=37 | 2.97 (0.50) N=58 | 0.471 |
|  | [2010,2017] | 2.79 (0.54) N=114 | 2.86 (0.49) N=202 | 0.471 |
| English |  | Speed Mean (sd) N | |  |
|  | Period of time | F | M | p |
|  | [1875,1960) | 2.26 (0.25) N=25 | 2.26 (0.41) N=55 | 0.982 |
|  | [1960,1980) | 2.81 (0.67) N=59 | 2.57 (0.52) N=212 | 0.006 |
|  | [1980,1990) | 3.02 (0.59) N=77 | 2.82 (0.64) N=176 | 0.039 |
|  | [1990,2000) | 2.98 (0.53) N=104 | 2.60 (0.57) N=160 | <0.001 |
|  | [2000,2010) | 2.81 (0.64) N=86 | 2.77 (0.63) N=184 | 0.748 |
|  | [2010,2017] | 2.82 (0.52) N=147 | 2.64 (0.46) N=322 | <0.001 |
| Manhattan |  | Speed Mean (sd) N | |  |
|  | Period of time | F | M | p |
|  | [1875,1960) ^2^ | 3.82 (0.77) N=6 | 4.92 (0.66) N=8 | 0.023 |
|  | [1960,1980) | 5.76 N=1 | - | - |
|  | [1980,1990) | 5.50 (0.58) N=54 | 5.31 (0.40) N=167 | 0.020 |
|  | [1990, 2000) | 6.04 (0.77) N=58 | 5.54 (0.54) N=165 | <0.001 |
|  | [2000,2010) | 5.43 (0.41) N=79 | 5.37 (0.40) N=139 | 0.401 |
|  | [2010,2017[ ^3^ | 5.41 (0.35) N=32 | 5.45 (0.31) N=65 | 0.555 |

^1^ Observations started in 1927, the first available calendar year for Catalina Channel Swim.

^2^ Observations started in 1915, the first available calendar year for Manhattan Island Marathon Swim.

^3^ Observations ended in 2012, the last available calendar year for Manhattan Island Marathon Swim.
